# Racial Differences in Prenatal Cannabis Screening Among Women at Elevated Risk for Substance Use and Sexually Transmitted Infections

**DOI:** 10.1111/jmwh.70105

**Published:** 2026-05-27

**Authors:** Kristina Countryman, Ananda Sen, Dongru Chen, Caron Zlotnick, Okeoma Mmeje, Katherine J. Gold, Golfo Tzilos Wernette

**Affiliations:** ^1^ Department of Family Medicine University of Michigan Medical School Ann Arbor Michigan; ^2^ Department of Biostatistics University of Michigan School of Public Health Ann Arbor Michigan; ^3^ Department of Psychiatry and Human Behavior Warren Alpert Medical School of Brown University Providence Rhode Island; ^4^ Department of Medicine Women and Infants Hospital Providence Rhode Island; ^5^ Department of Psychiatry and Mental Health University of Cape Town Groote Schuur Hospital Cape Town South Africa; ^6^ Department of Obstetrics and Gynecology University of Michigan Medical School Ann Arbor Michigan; ^7^ Department of Health Behavior and Health Education University of Michigan School of Public Health Ann Arbor Michigan

**Keywords:** breastfeeding, cannabis, pregnancy, racial bias, STIs

## Abstract

**Introduction:**

Cannabis use during pregnancy is rising, in part due to an increased perception of safety and legalization across the country. National guidelines recommend that clinicians universally screen for substance use, including cannabis use, during pregnancy.

**Methods:**

This is a longitudinal study assessing cannabis use frequency and reasons for use at baseline and patient‐clinician communication at 4 study timepoints. Data are drawn from a randomized controlled trial testing an innovative, technology‐delivered brief intervention, *Health Check‐up for Expectant Moms*, with 176 pregnant cisgender women (mean age, 30.2; SD, 5.02; 25.6% Black) reporting substance use risk, condomless sex, and multiple sexual partners. We used *t*‐tests, chi‐square tests, Fisher's exact tests, and logistic regression to analyze the data.

**Results:**

Overall, 18.2% (n = 32) self‐reported current cannabis use at baseline, and 26.1% (n = 46) reported past year use. The most cited reason for use was relaxation or tension relief. Black women were significantly more likely than White women to have reported being talked to by their clinician about cannabis use in general during pregnancy and breastfeeding at every timepoint (all timepoints *P* ≤ .01). Clustered logistic regression analysis also showed higher odds for Black women to have been talked to about cannabis use in general compared with White women (adjusted odds ratio, 4.09; 95% CI, 2.29‐7.32; *P* < .0001), and this significance prevailed even after adjusting for prior cannabis use.

**Discussion:**

Seeking relaxation or tension relief was the most cited reason for use during pregnancy. Results suggest a racial bias that led to significantly more Black women being asked about cannabis use during pregnancy or breastfeeding at every timepoint, as compared with White women. There is a need for systemic and clinician‐level improvement in universal screening and counseling.

## INTRODUCTION

Substance use and sexual risk behaviors are common and interlinked factors that contribute to adverse health outcomes, including among pregnant women.^1^ Cannabis is the most common federally illicit substance used in the United States and the most common illicit substance used during pregnancy.^2^ Cannabis use is on an upward trajectory during pregnancy, with a 62% increase from 2002 to 2014 in past‐month use reported during pregnancy.^3^ Recent data support these growing concerns; the 2022 National Survey on Drug Use and Health estimates of past‐month cannabis use among pregnant women aged 15 to 44 were 7.9% and 19.6% for nonpregnant women aged 15 to 44.^4^ In the changing US landscape of the legalization of recreational cannabis in recent years, there is a perception of the relative safety of cannabis use during pregnancy,^5^ Women report using it to help relieve common concerns in pregnancy: nausea, pain, sleep deprivation, and self‐medication of anxiety and depression.^6^ There is a clear association between substance use and sexual risk behavior, which increases the likelihood of acquiring sexually transmitted infections (STIs), especially in racial and ethnic minorities experiencing health disparities.^1^, ^7^, ^8^, ^9^, ^10^ STIs are of particular concern during pregnancy because they are associated with substantial morbidity and mortality, including miscarriage, fetal growth restriction (FGR), low birth weight (LBW), premature birth, and infant death.^11^

Cannabis use during pregnancy is associated with several adverse outcomes for the pregnant woman, including gestational hypertension and preeclampsia, placental abruption, gestational weight gain greater or less than established guidelines, and adverse mental health outcomes (eg, depression, panic disorder).^6^, ^12^ Regarding impact on the infant, cannabis use during pregnancy is associated with LBW, FGR, preterm birth, admission to the neonatal intensive care unit, and long‐term neural developmental issues affecting memory, learning, and behavior.^2^, ^13^, ^14^

The American College of Obstetricians and Gynecologists guidelines recommend universal screening of pregnant patients for alcohol, tobacco, cannabis, and illicit drugs using interviews, self‐reporting, or a well‐validated screening tool.^15^, ^16^ Despite this and the associated adverse neonatal health outcomes, routine clinician screening for cannabis use during pregnancy is often lacking and inconsistent.^5^, ^17^, ^18^ A 2019 study of obstetrician‐gynecologists’ attitudes and practices for substance use screening during pregnancy found that only 53% of clinicians considered cannabis screening a “high priority,” and only 10% reported using a validated screening tool for any substance use.^18^ Studies have identified numerous obstacles for clinician screening and communicating with pregnant patients about cannabis use, including discomfort with discussing risks due to insufficient and inconclusive data on the topic,^5^ lack of confidence in the ability to treat patients with substance use,^18^ feeling that their advice is unlikely to influence patient behavior,^19^ feeling overwhelmed with the amount of required screening assessments, limited clinical time, and assumptions about their patients’ use or nonuse of substances.^20^

Implicit racial biases, positive and negative subconscious beliefs and attitudes about different racial identities, and clinician assumptions influence health care delivery and outcomes.^21^ Because these biases are not within one's conscious awareness, they can be employed automatically, influencing one's behavior and clinical decision‐making.^22^ Clinicians are not immune to holding implicit racial biases, which contribute to disparities in health care outcomes in the United States.^23^ In one systematic review, Hall et al found that clinicians in 14 of 15 studies held low to moderate levels of implicit racial biases against Black, Hispanic and Latinx, and dark‐skinned people and that these biases were most significantly associated with poorer patient‐clinician interactions and negative health outcomes.^21^ Implicit biases also influence perinatal substance use screening, with multiple studies documenting how certain patient characteristics led to the clinician's decision on whether to screen (both verbal and biological) for substance use in the perinatal period.^24^, ^25^, ^26^, ^27^, ^28^, ^29^

**QUICK POINTS**

✦ We analyzed baseline trial data to describe cannabis use among pregnant women and how they experienced conversations about this use with their clinicians.
✦ Most pregnant women reported not having had any discussions with their clinicians about cannabis. Those who did have discussions were significantly more likely to be Black.
✦ The most cited reason for cannabis use during pregnancy was for relaxation or to relieve tension.
✦ This adds to the extant literature and aligns with other research demonstrating that stress, anxiety, and poor mental health are the top reasons for cannabis use during pregnancy.
✦ Our results extend previous findings that Black perinatal women are assessed for cannabis use more frequently than their White counterparts, despite national recommendations for universal screening.

The objectives of this study were to determine the frequency of and reasons for cannabis use during pregnancy and to understand the communication participants reported having with clinicians regarding cannabis use during pregnancy and breastfeeding. Our sample of pregnant women was at an increased risk for STIs and alcohol and drug use, and these participants were enrolled in the parent study.^30^

## METHODS

The current study is a longitudinal study of specific cannabis‐related data drawn from the parent study, a 2‐group, randomized controlled trial in which we recruited pregnant women meeting specific behavioral risk factors (including recent alcohol and drug use, condomless sex, multiple sexual partners, or a nonmonogamous partner). We randomized them to either (1) a web‐delivered, single session plus 2 booster brief intervention to reduce risky behaviors (Health Check‐up for Expectant Moms [HCEM]); or (2) a web‐delivered control condition. Data for each participant were collected for a period of up to 11 months. Recruitment, assessment, follow‐up procedures, and study aims have been described in detail previously.^30^, ^31^, ^32^

Participants were recruited in person and virtually between April 2019 and September 2023 from obstetrician‐gynecologist and family medicine clinics throughout a midwestern state. All participants went through an informed consent process, and the study protocol was initially approved by the University of Michigan Medical School Institutional Review Board (HUM00143896) on April 23, 2018, and is registered on ClinicalTrials.gov (NCT03826342). This study adheres to the Strengthening the Reporting of Observational Studies in Epidemiology reporting guidelines, and the checklist is provided in Supporting Information: Appendix S1.

### HCEM

HCEM is an innovative, low‐cost technology‐delivered intervention that targets the reduction of alcohol and drug use as well as STI risk behavior (eg, condomless sex, multiple partners) during pregnancy using a motivational interviewing approach.^33^ Theoretically grounded in the Information‐Motivation‐Behavior model,^34^ HCEM is a single session plus 2 brief sessions within one month of study enrollment. It was adapted during the COVID‐19 pandemic to be delivered entirely by remote participation.^31^

In this article, we present an examination of cannabis‐related data from the baseline, 2‐month, 6‐month, and 6‐week postpartum follow‐up assessments. We examined questions regarding the frequency of cannabis use, reasons for cannabis use (Table 2), and 2 questions regarding communications with clinicians surrounding cannabis use during pregnancy and breastfeeding. Specifically, “Has your doctor or nurse ever talked to you in general about cannabis use during pregnancy or breastfeeding?” and “Since being pregnant, has your doctor or nurse asked you about your cannabis use?” These questions were web‐based and self‐reported, similar to the demographic questions.

### Baseline Assessments

All study assessments used standard and validated measures for this population (eg, the Alcohol Use Disorders Identification Test, Timeline Follow Back, Condom Attitude Scale).^35^, ^36^, ^37^ Participants completed the web‐based baseline assessment in person at the clinic with an iPad or at home through our secure, Health Insurance Portability and Accountability Act–compliant study website using a computer or mobile device. Consistent with the literature, the Timeline Follow Back (a validated and reliable calendar‐based assessment in which self‐reported, daily substance use and sexual behavior is tracked) was conducted either in person or by telephone via interview with a research assistant. Baseline assessments were conducted early in the participants’ pregnancies (≤21 weeks) and before the receipt of either the intervention or control condition. Gestational age was self‐reported and validated via the health record. The baseline assessment visit included self‐reported questions on demographics (Table 1) and behaviors and attitudes related to our trial objectives.

**Table 1:** Participant Demographics (N = 176)

Characteristic	Value
**Age, mean (SD), y**	30.17 (5.02)
**Pregnancy wk, mean (SD)**	13.8 (4.4)
**Race, n (%)**	
White	112 (63.6)
American Indian or Native Alaskan	1 (0.6)
Black	45 (25.6)
Asian	4 (2.3)
Biracial	7 (4.0)
Other	7 (4.0)
**Ethnicity, n (%)**	
Latina	17 (9.7)
Non‐Latina	159 (90.3)
**Current marital status, n (%)**	
Single and never married	41 (23.3)
Living together but not married	33 (18.8)
Legally married	95 (54.0)
Separated	2 (1.1)
Divorced	4 (2.3)
Widowed	1 (0.6)
**Education, n (%)**	
Did not graduate high school	11 (6.3)
High school graduate or GED	23 (13.1)
Went to technical or trade school	8 (4.6)
Some college	33 (18.8)
College graduate	49 (27.8)
Postgraduate	52 (29.6)
**Employment status, n (%)**	
Full time	99 (56.3)
Part time	27 (15.3)
Student	5 (2.8)
Stay‐at‐home mom	22 (12.5)
Unemployed	23 (13.1)
**Income, n (%)**	
<$50,000	76 (43.2)
≥$50,000	100 (56.8)
**Receive public assistance? n (%)**	
Yes	56 (31.8)
No	120 (68.2)

Abbreviation: GED, General Educational Development.

### Follow‐up Assessments

Participants completed the same web‐based follow‐up assessments at 2 months and 6 months postintervention or control condition and again at 6 weeks postpartum. The Timeline Follow Back interview with the research assistant was also completed at each follow‐up visit. However, the web‐based questions regarding reasons and frequency of cannabis use were only assessed at the baseline assessment, whereas the 2 questions regarding clinician discussions surrounding cannabis use during pregnancy and breastfeeding were asked at every follow‐up assessment.

### Data Analysis

All analyses were conducted using SAS software, version 9.4 (SAS Institute Inc, Cary, NC, USA). Key study variables and participant characteristics were summarized through descriptive statistics. To avoid sparsity, some categories in marital status, education, income, and employment status were collapsed before conducting any comparative analysis. Racial differences in socioeconomic status, as well as other demographic variables, and clinician communications about cannabis use were investigated using *t*‐tests, chi‐square tests, or Fisher's exact tests, as appropriate. In any analysis involving race, we did not adjust for any of the sociodemographic variables, allowing us to assess the unmasked effect of race. Clinician communications about cannabis use and race over time were investigated using a clustered logistic regression model with race and a 4‐category time variable (baseline, 2 months, 6 months, and 6 weeks postpartum) via a generalized estimating equations approach. To control for potential confounding, a sensitivity analysis was carried out by further adjusting the regression model for the self‐reported response to the question of prior cannabis use at baseline.

### Reflexivity Statement

The first author of this study identifies as a White woman, has training in public health, and is an advocate for health equity, antiracist scholarship, and racial justice. She has been influenced by the work of Camara Phylis Jones and Chandra Ford. The senior author of this study identifies as a White woman and is a behavioral scientist with a focus on the prevention of substance use and related health behaviors, primarily in women; she was also the principal investigator of the study. The analysis was conducted by the second and third authors, statisticians in our department. The first author was involved in participant recruitment and data collection and, together with the last author and statisticians, conceived the research design for this article. The fourth, fifth, and sixth authors were co‐investigators on the study and offered their expertise and review of the research design, data interpretation, and the overall writing of the article. A co‐author of this study identifies as a White woman and has spent much of her career developing and testing interventions for underserved and underresearched populations, including perinatal women on public assistance, incarcerated women with HIV risk and using substances, and women experiencing intimate partner violence. She has also published articles on mentoring individuals from diverse backgrounds.

## RESULTS

The full study sample included a total of 176 pregnant cisgender women between the ages of 18 to 45 and ≤21 weeks’ gestation (mean age, 30.2; SD, 5.02; 25.6% Black; see Table 1). At baseline, almost 70 percent (67%) of our sample self‐reported past cannabis use, ranging from more than 12 months ago to within the last 30 days (Table 2). A total of 18.2% reported current use (within the last 30 days), and 26.1% reported use within the last 12 months. The 2 most cited reasons for cannabis use were to relax and relieve tension (52.9%) and to help with sleep (45.4%) (Table 2). Race introduced a significant imbalance in the data. In our sample, Black women reported significantly less education, lower income, and a lower propensity for being full‐time or part‐time employed, as well as a higher propensity for receiving public assistance (see Table 3). Curiously, none of the Black women in our sample were legally married, whereas 78.6% of the White women were in a marital relationship at the time of the study.

**Table 2:** Frequency of Cannabis Use and Reasons for Use at Baseline (N = 176)

Cannabis Use	Value
**How long has it been since you last used marijuana? n (%)**	
I have not used marijuana or hashish	58 (33.0)
Within the past 30 days	32 (18.2)
More than 30 days ago but within the past 12 mo	46 (26.1)
More than 12 mo ago	40 (22.7)
**What are reasons that you used marijuana? (Select all that apply), n/N (%)**	
To relieve physical pain	33/118 (28.0)
To relax or relieve tension	63/118 (53.4)
To experiment or see what it is like	23/118 (19.5)
To feel good or get high	51/118 (43.2)
To help with my sleep	54/118 (45.8)
To help me with my feelings or emotions	31/118 (26.3)
To increase or decrease the effect(s) of some other drug	1/118 (0.8)
Because I am “hooked” or I have to have it for some other reason	3/118 (2.5)
To relieve nausea and vomiting	25/118 (21.2)
Do not know or other reason	4/118 (3.4)
Never asked or not applicable	58/176 (33)

**Table 3:** Demographic Comparisons by White and Black Participants (N = 157)

Demographics	White (n = 112)	Black (n = 45)	*P* Value^a^
**Age, mean (SD)**	30.8 (4.6)	29.0 (5.4)	.04
**Pregnancy wk, mean (SD)**	13.9 (4.6)	13.6 (4.2)	.65
**Ethnicity, n (%)**			.73
Latina	9 (8.0)	2 (4.4)	
Non‐Latina	103 (92.0)	43 (95.6)	
**Current marital status, n (%)**			<.0001
Legally married	88 (78.6)	0 (0)	
Living together but not married	12 (10.7)	16 (35.6)	
Single and never married, separated, divorced, or widowed	12 (10.7)	29 (64.4)	
**Education, n (%)**			<.0001
Less than eighth grade or HS diploma or GED	9 (8.0)	19 (42.2)	
Technical or trade school or some college	21 (18.8)	15 (33.3)	
College graduate or postgraduate	82 (73.2)	11 (24.4)	
**Income, n (%)**			<.0001
<$50,000	26 (23.2)	40 (88.9)	
≥$50,000	86 (76.8)	5 (11.1)	
**Employment status, n (%)**			.005
Full time	70 (62.5)	21 (46.7)	
Part time	20 (17.9)	4 (8.9)	
Student, homemaker, or unemployed	22 (19.6)	20 (44.4)	
**Receive public assistance? n (%)**			<.0001
Yes	17 (15.2)	30 (66.7)	
No	95 (84.8)	15 (33.3)	

Abbreviations: GED, general education development; HS, high school.

a Differences between races were compared using *t*‐tests, chi‐square tests, or Fisher's exact tests, as appropriate.

**Figure 1:**
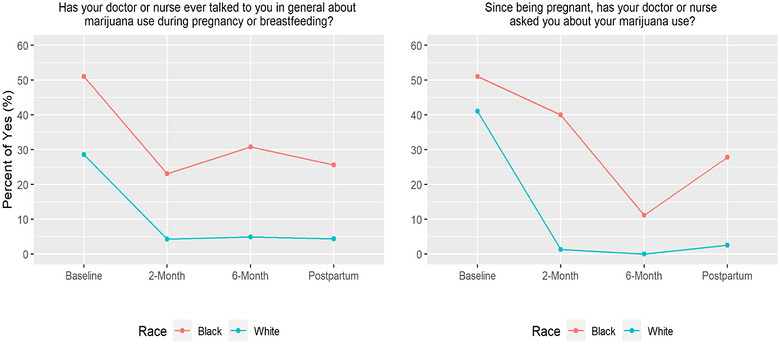
Comparison of the Percentage of “Yes” Responses by Race at Each Study Timepoint to Selected Questions

We were primarily interested in clinician communication about cannabis use during pregnancy and breastfeeding. At baseline (N = 176), when asked if their doctor or nurse had ever talked to them in general about cannabis use during pregnancy or breastfeeding, 64.8% (n = 114) of participants said “no.” Similarly, most participants (55.11%; n = 97) said “no” when asked if their doctor or nurse had ever asked them specifically about their own cannabis use since being pregnant. This trend continued throughout the study, with fewer conversations reported at each follow‐up assessment. Further analyzing these communications by race (ie, White women and Black women; n = 157), we found significant racial differences in the proportion of women that reported having been asked or talked to by their clinician about cannabis use in general during pregnancy or breastfeeding at baseline, with Black women being significantly more likely than White women to have been asked or talked to by their clinicians (51% vs 29%; *P* = .007). This significance was observed at every study follow‐up timepoint (see Table 4 and Figure 1). Exhibiting a similar trend, Black women were more likely than White women to report being asked specifically about their own cannabis use at every study timepoint. However, this trend was only significant at the 2 months and 6 weeks postpartum timepoints (see Table 4).

**Table 4:** Experience with Clinician Counseling on Cannabis Use During Pregnancy by Race and Study Timepoint

Questions Regarding Communications With Clinicians (n = sample size at each study assessment time point)	All, n (%)		White, n (%)		Black, n (%)		
	Yes	No	Yes	No	Yes	No	*P* Value^a^
**Has your doctor or nurse ever talked to you in general about marijuana use during pregnancy or breastfeeding?**							
Baseline (n = 157)	55 (35.0)	102 (65.0)	32 (28.6)	80 (71.4)	23 (51.0)	22 (49.0)	.0074
2 mo (n = 133)	13 (9.8)	120 (90.2)	4 (4.3)	90 (95.7)	9 (23.1)	30 (76.9)	.0021
6 mo (n = 87)	11 (12.6)	76 (87.4)	3 (4.9)	58 (95.1)	8 (30.8)	18 (69.2)	.0022
6 wk postpartum (n = 130)	14 (10.8)	116 (89.2)	4 (4.4)	87 (95.6)	10 (25.6)	29 (74.4)	<.001
**Since being pregnant, has your doctor or nurse asked you about your marijuana use?**							
Baseline (n = 157)	69 (43.9)	88 (56.1)	46 (41.1)	66 (58.9)	23 (51.0)	22 (49.0)	.2518
2 mo (n = 90)	7 (7.8)	83 (92.2)	1 (1.3)	74 (98.7)	9 (60.0)	6 (40.0)	<.0001
6 mo (n = 57)	1 (1.8)	56 (98.2)	0	48 (100%)	1 (11.0)	8 (89.0)	.1579
6‐wk postpartum (n = 96)	7 (7.3)	89 (92.7)	2 (2.6)	76 (97.4)	5 (27.8)	13 (72.2)	.0023

a *P* values were obtained using chi‐square or Fisher's exact tests as appropriate.

Clustered logistic regression analysis showed higher odds for Black women to have been asked or talked to about cannabis use in general by their clinicians compared with their White counterparts (adjusted odds ratio [aOR], 4.09; 95% CI, 2.29‐7.32; *P* < .0001). After adjusting for the racial disparity, overall, the later timepoints showed significantly lower odds compared with baseline in the likelihood of clinicians having talked to them about cannabis use in general (see Table 5). With regard to asking about their own cannabis use, Black women had significantly higher odds of being asked than their White counterparts (aOR, 2.47; 95% CI, 1.2‐5.11; *P* = .014). Similar to the previous item, all postbaseline timepoints had significantly lower odds of being asked about their own cannabis use (Table 5), although the odds ratios were associated with large CIs due to the low prevalence of women who said “yes” to the question. In a sensitivity analysis (Table 6) that controlled the regression model for prior cannabis use, the significance of the Black‐White disparity prevailed (aOR, 3.61; 95% CI, 2.01‐6.49; *P* < .0001).

**Table 5:** Adjusted Odds Ratios and 95% CIs Based on a Clustered Logistic Regression With a Generalized Estimating Equations Approach for Selected Questions

	Has Your Doctor or Nurse Ever Talked to You in General About Marijuana Use During Pregnancy or Breastfeeding?		Since Being Pregnant, Has Your Doctor or Nurse Asked You About Your Marijuana Use?	
Variables	aOR (95% CI)^a^	*P* Value	aOR (95% CI)^a^	*P* Value
**Race**				
White	1 [Reference]		1 [Reference]	
Black	4.09 (2.29‐7.32)	<.0001	2.47 (1.20‐5.11)	.0143
**Time**				
Baseline	1 [Reference]		1 [Reference]	
2 mo	0.26 (0.14‐0.50)	<.0001	0.16 (0.08‐0.34)	<.0001
6 mo	0.19 (0.09‐0.38)	<.0001	0.02 (0.01‐0.11)	<.0001
Postpartum	0.37 (0.20‐0.70)	.0019	0.21 (0.09‐0.45)	<.0001

Abbreviation: aOR, adjusted odds ratio.

a Logistic regression models adjusted for time.

**Table 6:** Adjusted Odds Ratios and 95% CIs Based on a Clustered Logistic Regression With a Generalized Estimating Equations Approach After Controlling for Prior Cannabis Use for Selected Questions

	Has Your Doctor or Nurse Ever Talked to You in General About Marijuana Use During Pregnancy or Breastfeeding?		Since Being Pregnant, Has Your Doctor or Nurse Asked You About Your Marijuana Use?	
Variables	aOR (95% CI)	*P* Value	aOR (95% CI)	*P* Value
**Race**				
White	1 [Reference]		1 [Reference]	
Black	3.61 (2.01‐6.49)	<.0001	2.17 (1.03‐4.56)	.0407
**Ever smoked marijuana**				
No	1 [Reference]		1 [Reference]	
Yes	2.66 (1.32‐5.34)	.0061	2.62 (1.31‐5.21)	.0062
**Time**				
Baseline	1 [Reference]		1 [Reference]	
2 mo	0.25 (0.13‐0.49)	<.0001	0.16 (0.07‐0.33)	<.0001
6 mo	0.18 (0.09‐0.37)	<.0001	0.02 (0‐0.10)	<.0001
Postpartum	0.36 (0.19‐0.68)	.0016	0.19 (0.09‐0.43)	<.0001

Abbreviation: aOR, adjusted odds ratio.

a Logistic regression models adjusted for time.

## DISCUSSION

In this study, we found that the reasons for cannabis use during pregnancy are similar to those reported in previous research, with wanting to relax or relieve tension and to help with sleep being the 2 most frequently cited reasons. Self‐reported cannabis use within the last 30 days was 18.2% in our sample. At every study timepoint, most participants denied any communication with their clinicians about cannabis use during pregnancy or breastfeeding, but for those who did report positively, they were significantly more likely to be Black women rather than White women.

Several recent qualitative studies reported participants using cannabis during pregnancy for medicinal purposes, namely to relieve anxiety, stress, depression, pain, and nausea.^38^, ^39^, ^40^ Similarly, in an analysis of the Pregnancy Risk Assessment Monitoring System (PRAMS) data from 8 states, Ko et al found that the most cited reasons for cannabis use during pregnancy were to relieve stress or anxiety (81.5%), nausea and vomiting (77.8%), and pain (55.1%).^41^ A 2022 population‐based survey of recently pregnant women across the United States mirrors those found in the PRAMS study: 81% of women reported using cannabis during pregnancy to relieve anxiety and 80% to relieve nausea.^42^ Taken together, these findings suggest a persistent unmet need among women for stress reduction tools, mental health care, and safer alternatives for physical health symptom relief (eg, nausea, pain) during pregnancy.

Prior interviews have found that pregnant women using cannabis desire medical information from their clinicians about the potential effects of cannabis use on their infants.^43^ Considering this and the national screening guidelines, our findings are concerning. The majority of our participants denied any communications with their clinician about cannabis use during pregnancy and breastfeeding, which is higher than national data report. Previous work revealed that approximately one‐third of women were not asked about their cannabis use at any of their prenatal visits during their most recent pregnancy.^44^ A lack of communication from clinicians about the health risks of cannabis use during pregnancy can be interpreted by pregnant women as there being limited to no potential harms associated with cannabis use.^5^, ^43^ Furthermore, national data reveal that women who report cannabis use during pregnancy are also more likely to report other substance use as well as major depression and other serious mental illnesses.^45^, ^46^, ^47^ One such study found that women reporting prenatal depression or anxiety were 2 to 4 times more likely to also report cannabis use.^48^ Avoiding discussions about cannabis use during the perinatal period denotes a missed opportunity for clinicians to potentially identify other substance use (eg, alcohol, tobacco) and mental health concerns during a critical time.

Our findings that Black women were significantly more likely than White women to report having been asked or talked to by their clinician about cannabis use during pregnancy or breastfeeding are disquieting. Prior research has identified similar racial and demographic heterogeneity for substance use screening during pregnancy. Pregnant women were more likely to receive substance use screening if they were Black, single and unmarried, younger in age, publicly insured, had less prenatal care, had a history of cigarette and illicit drug use, had a history of being reported or investigated by Child Protective Services, had a placental abruption, had a social risk factor (eg, financial difficulty, family violence), or had a mental health condition.^24^, ^25^ Although several of these factors that led to substance use screening are medically justifiable (eg, history of cigarettes or substance use), determining who is asked solely based on racial and/or ethnic background or insurance type is not justifiable and suggests a biased approach to screening. Furthermore, this screening bias has recently been shown in a study on clinician‐ordered meconium drug screening for newborns of Black birthing people in a large, midwestern US hospital system.^29^ And although national guidelines recommend that clinicians counsel against using cannabis while breastfeeding, they do not recommend counseling against breastfeeding because of cannabis use.^17^, ^49^ Prior work has found that despite this recommendation, non‐Hispanic Black pregnant women reporting cannabis use were 4 times more likely to be advised against breastfeeding than non‐Hispanic White pregnant women also reporting cannabis use.^49^

Our findings demonstrate a clear need to improve the care that is provided to pregnant women concerning communications around cannabis use. Health care clinicians play an important role in educating and helping pregnant women navigate their understanding of health risks, especially when they are unsure about the scope of those risks.^5^ There has been considerable research in recent years regarding acceptable, nonjudgmental, validated, and reliable screening tools for alcohol and other drug use during pregnancy.^50^, ^51^, ^52^ A prior study of pregnant women found that most (97%) found it acceptable to disclose and discuss their substance use with clinicians during prenatal care.^53^ At the same time, internalized stigma and fears of being stigmatized by clinicians can also lead pregnant women to delay disclosure of substance use and delay or avoid prenatal care altogether.^46^ Equipped with the knowledge that pregnant women and prenatal clinicians have found screening to be acceptable, it is critical to recognize that stigma, clinician biases, and pregnant women's perceptions of adverse legal consequences (eg, for themselves and their infant),^40^ are significant barriers for honest communication and, therefore, optimal health during pregnancy.

The question then is, how do we best achieve equity in cannabis screening and communication? Most mitigation responses to date have used implicit bias training (IBT) for clinicians; however, the evidence for the efficacy of IBT on improving patient outcomes and clinical practice is scarce.^54^, ^55^ At best, research on IBT has shown modest improvements in individual awareness of implicit bias and motivations to address these biases, and the improvements have been short‐term in effect.^54^, ^55^ Noting this limitation, Siden et al put forth an action framework to reduce implicit bias in maternity care that expands beyond traditional IBT. Development of any implicit bias intervention needs to prioritize patient perspectives (eg, patient advisory boards) and include the following 3 domains deemed “critical” for success: (1) education and self‐awareness, (2) communication skills, and (3) cognitive reframing.^55^ The need for these components was also found in the qualitative work of Garrett et al, particularly the need to design implicit bias interventions with the ongoing feedback of those most affected—the patients themselves.^54^

Moreover, it is imperative to look for opportunities to enhance and support providers’ ability and confidence in conducting and implementing routine screening and providing recommendations to discontinue substance use.^18^ For example, this can be improved by enhancing education programs to circulate validated tools and train providers and staff who care for pregnant and postpartum individuals.^5^, ^18^ Additionally, implementing procedural changes to include validated screening tools at every prenatal visit would also allow time for a trusting patient‐provider relationship to develop, potentially mitigating fears of disclosure. Making such screening routine for every prenatal visit could also help normalize the practice, which could further mitigate fears and stigma.

The strengths of this study include a broad assessment of cannabis‐related concerns among our sample of pregnant women with behavioral health risks. The assessments analyzed in this study were validated measures and were web‐delivered and, therefore, possibly facilitated disclosure. A limitation, however, is that our sample was recruited from a midwestern state with legalized recreational cannabis, and our results may not be generalizable to all pregnant women. Our sample included only cisgender women who were at an increased risk for STIs during pregnancy based on specific sexual health behaviors and, therefore, may not be generalizable to all pregnant individuals. Furthermore, there were differences in the number of participants completing follow‐up visits, which also limits the generalizability of the results. Finally, we did not conduct open‐ended questions or retrospective interviews to understand more about the reported participant‐clinician communications surrounding cannabis use during pregnancy and breastfeeding. It is possible that participants reporting communications about cannabis use may have been the ones to bring up the subject, not their clinicians. Because we do not know exactly what was discussed, we cannot determine whether the participants perceived these as positive or negative communications. Inadequate qualitative data to better understand screening practices for cannabis use during pregnancy is also noted as a limitation in prior work. One recent study sought to address this gap by completing qualitative interviews with predominantly Black postpartum women who recently gave birth in a US hospital. It collected important data on perceptions of cannabis use, reasons for cannabis use, and the barriers and facilitators to cannabis use disclosure, including fears of punitive consequences (barrier) and perceived positive clinician communication (facilitator).^38^ More qualitative work like this is needed for both pregnant women and clinicians so we can dismantle the barriers and elevate the facilitators effectively.

## CONCLUSION

In the current study, we characterize the experiences of cannabis use in our sample of pregnant women at risk for substance use and STIs. This study makes a unique contribution to the literature by reporting on the impact of racial status on clinician communications surrounding cannabis use during pregnancy and breastfeeding. Our results suggest a racial bias that led to significantly more Black women being asked about cannabis use during pregnancy or breastfeeding, as compared with White women at every study timepoint, even when adjusting for prior cannabis use. Based on this work and previous studies that have demonstrated racial differences in the context of perinatal care provision, we agree that there is a need for systemic and clinician‐level improvements in both universal screening and counseling, as per national guidelines. Furthermore, there is a need to include strategies to address barriers to universal screening and counseling. For example, IBT to minimize stigmatization during the perinatal period, education programs to train providers and staff on successful routine screening techniques and intervention tools, and the optimal design of these trainings is iterative and patient centered.

## CONFLICT OF INTEREST

The authors have no conflicts of interest to disclose.

## Supporting information




**Appendix S1**. Strengthening the Reporting of Observational Studies in Epidemiology (STROBE) checklist.
